# Artificial MicroRNAs as Novel Secreted Reporters for Cell Monitoring in Living Subjects

**DOI:** 10.1371/journal.pone.0159369

**Published:** 2016-07-21

**Authors:** John A. Ronald, Aloma L. D’Souza, Hui-Yen Chuang, Sanjiv Sam Gambhir

**Affiliations:** 1 Molecular Imaging Program at Stanford, Stanford University, Stanford, California, United States of America; 2 Department of Radiology, Stanford University, Stanford, California, United States of America; 3 Department of Biomedical Imaging and Radiological Sciences, National Yang-Ming University, Taipei, Taiwan; IRCCS-Policlinico San Donato, ITALY

## Abstract

Reporter genes are powerful technologies that can be used to directly inform on the fate of transplanted cells in living subjects. Imaging reporter genes are often employed to quantify cell number, location(s), and viability with various imaging modalities. To complement this, reporters that are secreted from cells can provide a low-cost, *in vitro* diagnostic test to monitor overall cell viability at relatively high frequency without knowing the locations of all cells. Whereas protein-based secretable reporters have been developed, an RNA-based reporter detectable with amplification inherent PCR-based assays has not been previously described. MicroRNAs (miRNAs) are short non-coding RNAs (18–22 nt) that regulate mRNA translation and are being explored as relatively stable blood-based disease biomarkers. We developed an artificial miRNA-based secreted reporter, called Sec-miR, utilizing a coding sequence that is not expressed endogenously and does not have any known vertebrate target. Sec-miR was detectable in both the cells and culture media of transiently transfected cells. Cells stably expressing Sec-miR also reliably secreted it into the culture media. Mice implanted with parental HeLa cells or HeLa cells expressing both Sec-miR and the bioluminescence imaging (BLI) reporter gene Firefly luciferase (FLuc) were monitored over time for tumor volume, FLuc signal via BLI, and blood levels of Sec-miR. Significantly (p<0.05) higher Sec-miR was found in the blood of mice bearing Sec-miR-expressing tumors compared to parental cell tumors at 21 and 28 days after implantation. Importantly, blood Sec-miR reporter levels after day 21 showed a trend towards correlation with tumor volume (R^2^ = 0.6090; p = 0.0671) and significantly correlated with FLuc signal (R^2^ = 0.7067; p<0.05). Finally, we could significantly (p<0.01) amplify Sec-miR secretion into the cell media by chaining together multiple Sec-miR copies (4 instead of 1 or 2) within an expression cassette. Overall, we show that a novel complement of BLI together with a unique Sec-miR reporter adds an *in vitro* RNA-based diagnostic to enhance the monitoring of transplanted cells. While Sec-miR was not as sensitive as BLI for monitoring cell number, it may be more sensitive than clinically-relevant positron emission tomography (PET) reporter assays. Future work will focus on improving cell detectability via improved secretion of Sec-miR reporters from cells and more sensitive detection platforms, as well as, exploring other miRNA sequences to allow multiplexed monitoring of more than one cell population at a time. Continued development may lead to more refined and precise monitoring of cell-based therapies.

## Introduction

Precise tracking of cell-based therapies (e.g., stem cells, immune cells, etc.) can become a reality if technologies for measuring transplanted cell numbers, location(s), viability, and cell status are utilized in the clinic [1]. This could allow clinicians to directly monitor therapeutic effectiveness in individual patients and give information on both subsequent treatment decisions and a patient’s overall prognosis. An exciting prospect is to engineer cells to stably express imaging reporter genes prior to transplantation, which allows one to serially monitor their fate with non-invasive molecular imaging. Many imaging reporters now exist for use at both the pre-clinical level such as Firefly luciferase (FLuc) and/or Renilla Luciferase (Rluc) for bioluminescence imaging (BLI) [2–4], or various reporters for clinical modalities such as magnetic resonance imaging (MRI) [5–7], single photon emission computed tomography (SPECT) [8], and positron emission tomography (PET) [9, 10]. Recently our group has demonstrated the first use of PET reporter genes for tracking cytotoxic T cell cancer immunotherapy in patients [11], highlighting the translational potential of these state-of-the-art reporter technologies.

While imaging can provide critical information regarding cell location(s) and viability, two fundamental limitations of an imaging strategy is the frequency that a patient can be imaged, arising from both safety concerns and the financial costs associated with each imaging session, and the sensitivity to detect small numbers of cells. Limit estimates with a clinical PET scanner include ~100x10^6^ human mesenchymal stem cells injected into porcine myocardium [12]. One solution to these issues is to combine an imaging reporter assay with a relatively cheap and sensitive *in vitro* blood-based reporter assay. This allows the use of the blood test to assess whole-body overall survival of the transplanted cells at regular intervals, in addition to, less frequent imaging sessions to visualize the location(s) and number of cells. Costly imaging would be employed particularly if a change in cell status was detected in the blood assay. This combined *in vivo* imaging with an *in vitro* diagnostic reporter gene strategy is gaining popularity among those developing new reporter gene technologies [13, 14], and has recently been utilized in several gene-based cancer detection technologies in small animals [15–18].

Several secreted reporter proteins have been described including soluble marker peptides derived from human chorionic gonadotropin and human carcinoembryonic antigen [19], secreted alkaline phosphatase (SEAP) [20], and *Gaussia* luciferase (GLuc) [14], amongst others [21]. According to Tannous and Teng [21], the ideal characteristics of a secreted reporter gene would include: 1) minimal endogenous expression from normal tissues; 2) stable expression in immunocompetent animals (lack of an immunological response); and 3) rapid, sensitive and specific detection. Quantitative-real-time PCR (qRT-PCR) is a simple, standardized assay for quantitation of RNA and is highly sensitive (inherent amplification of signal), highly specific, and reproducible. Thus in terms of assay sensitivity and specificity, an RNA-based reporter gene could have many advantages. However, to our knowledge, an RNA-based secreted reporter has not been previously described.

MicroRNAs (miRNA) are a large class of highly conserved, short (~22 nucleotides), non-coding RNAs that mediate post-transcriptional regulation by binding to and repressing specific target mRNAs [22, 23]. In humans, approximately 30% of mRNAs are regulated by miRNAs and each miRNA is able to regulate upwards of hundreds of mRNAs [24, 25]. miRNAs are also detectable in a cell-free form within the circulatory system and their levels are often altered in cancer and other diseases [26, 27]. Remarkably, despite the high level of RNAse activity in the blood, miRNAs are resistant to degradation, can be readily measured in both plasma and serum to differentiate tumor-bearing and healthy subjects in both animal models and patients, and are being extensively explored as a new class of blood-based disease biomarkers [27–31]. This stability appears to be regulated by several mechanisms including encapsulation in membrane-bound vesicles such as exosomes (50–90 nm in size) [32–34] and microvesicles (up to 1 mm in size) [35], but also being released bound to the ribonucleoprotein Argonaute2, a core component of the miRNA-induced silencing complex [36]. Due to their ability to be released by cancer cells into the circulation and their high blood stability we hypothesized that miRNAs can be utilized as a novel class of blood-based reporter genes.

Rather than using an endogenous miRNA as a reporter gene, we utilized an artificial or secreted miRNA (Sec-miR; AAAUGUACUGCGCGUGGAGAC) that is not normally expressed in vertebrates and whose sequence has been predicted not to bind any known vertebrate gene. Hence, we do not expect any background expression in animals and induced expression of our Sec-miR reporter should have no known unwanted biological effects. In addition, the Sec-miR sequence contains a GGAG EXOmotif in the 3’ half of the miRNA sequence, which has been implicated in helping load miRNAs into exosomes [37]. Here we validated the ability to detect our Sec-miR reporter both *in cell culture* and *in vivo* in living mice, and further confirmed the ability to utilize our blood-based Sec-miR reporter as a complementary tool to *in vivo* imaging reporters for monitoring the growth of proliferating cell populations in living subjects. Continued development of this novel secreted reporter technology may enable integrated *in vitro* and imaging diagnostics to be used for monitoring cell-based therapeutics in both small animal models and eventually in patients.

## Materials and Methods

### Ethics Statement

The Administrative Panel on Laboratory Animal Care at Stanford University approved all animal experiments and all efforts were made to minimize animal suffering.

### Vector Construction

The original construct, pcDNA 6.2-GW/EmGFP-miR-neg, was purchased from a commercial vendor (Life Technologies). This vector is driven by the cytomegalovirus promoter (pCMV) and encodes Emerald Green Fluorescent Protein (EmGFP) and a pre-miRNA sequence called miR-neg in the 3’ untranslated region (UTR). miR-neg is composed of the Sec-miR sequence flanked by murine miR-155 sequences to ensure proper processing of the pre-miRNA to mature Sec-miR (AAAUGUACUGCGCGUGGAGAC) [38]. This construct is typically used as a negative control for miRNA overexpression experiments. We replaced EmGFP with the codon-optimized bioluminescence imaging (BLI) reporter gene Firefly luciferase (Luc2—gene; FLuc—protein) to generate the pcDNA 6.2-GW/pCMV-Luc2-miR-neg. In addition, to avoid silencing associated with pCMV in stable cells, we replaced pCMV in pcDNA 6.2-GW/pCMV-Luc2-miR-neg with the elongation factor-1 alpha promoter (pEF1) to generate pcDNA 6.2-GW/pEF1-Luc2-miR-neg. We also made constructs that expressed 2 or 4 copies of Sec-miR (pcDNA 6.2-GW/pCMV-Luc2-miR-negx2 and pcDNA 6.2-GW/pCMV-Luc2-miR-negx4, respectively), according to the manufacturer’s instructions (Life Technologies). Briefly, the miR-neg pre-miRNA insert was obtained by digesting pcDNA 6.2-GW/pCMV-Luc2-miR-neg with *Bam HI* and *Xho I*. This was then cloned downstream of miR-neg in pcDNA 6.2-GW/pCMV-Luc2-miR-neg after digestion with *Bgl II* and *Xho I* to generate pcDNA 6.2-GW/pCMV-Luc2-miR-negx2. To generate pcDNA 6.2-GW/pCMV-Luc2-miR-negx4 we performed the exact same procedures but used pcDNA 6.2-GW/pCMV-Luc2-Sec-miRx2 as both the insert donor (neg-miRx2 pre-miRNA) and acceptor. All constructs were amplified in Top10 E. coli (Life Technologies) and purified using endotoxin-free maxi kits (Qiagen, Valencia, CA).

### Cell Culture

HeLa (human cervical adenocarcinoma; American Tissue Culture Collection (ATCC)) cells were maintained at 37°C in a humidified atmosphere containing 5% CO_2_. Cells were grown in DMEM high glucose medium (Gibco, Carlsbad, CA) supplemented with 10% fetal bovine serum (FBS), 1x non-essential amino acids, and an antibiotic-antimycotic solution (ThermoFisher; 100 units/mL of penicillin, 100 μg/mL of streptomycin, 0.25 μg/mL of Gibco Amphotericin B).

### Transient Transfection and Stable Cell Generation

For transient transfections, we plated 2x10^6^ cells in 10 cm dishes one day prior to transfection. Cells were transfected with the various constructs (10 μg) using a polyethylenimine transfection agent (20 μl; jet-PEI; PolyPlus Transfection), according to the manufacturer’s instructions. Cells were washed 24 hours after transfection, and cells and media was collected 48 hours after washing. Phase and fluorescence microscopy (EmGFP) images of cells transfected with pcDNA 6.2-GW/EmGFP-miR-neg were collected on an EVOS FL microscope (Life Technologies). The number of cells that were EmGFP-positive were counted in two separate fields of view to assess transfection efficiency. For stable cell generation, we transiently transfected cells with pcDNA 6.2-GW/pEF1-Luc2-miR-neg and 24 hours later added blasticidin (20 μg/ml; Life Technologies). One week later we plated ~100 cells into 10 cm dishes, grew these for ~1–2 weeks, isolated clonal populations expressing FLuc (as visualized via BLI) using cloning rings, and expanded these cell populations. Nine clonal populations were screened for FLuc activity levels by measuring light output (relative light units; RLU) on a luminometer (Turner 20/20) after an addition of LAR-II reagent (10 second integration time). RLU levels were normalized to protein content using a Pierce 660 protein assay and the clonal population with the highest RLU/μg protein levels, called Sec-miR/FLuc, was used for all subsequent experiments.

### Mouse Studies

We implanted female Nu/Nu mice with 2x10^6^ parental HeLa (n = 5) or Sec-miR/FLuc (n = 5) HeLa cells into the flank of each mouse. At weekly intervals, calipers were used to evaluate tumor volume (mm^3^; width^2^ x length x 4/3π), and BLI was performed at 1-minute intervals from 5 to 20 minutes after intraperitoneal injection of D-Luciferin (30 mg/ml; 3 mg total dose) using an IVIS Spectrum (PerkinElmer). At endpoint, mice were sacrificed via CO_2_ inhalation followed by cervical dislocation. In each BLI image regions of interest (ROI) were drawn over the tumor area, and peak FLuc signal over the 15 minutes of scan time was used as the measure of viable Sec-miR/FLuc cells. Plasma samples were taken from submandibular bleeds from the mice.

### miRNA Expression Analysis

We evaluated miRNA levels in cells, media, and plasma following previously published methods [39]. Total RNA including miRNA was extracted from cultured cells using the miRNeasy Mini kit (Qiagen). For media, to avoid cells and cellular debris we spun media samples at 2000 RPM for 5 minutes followed by passing it through a 0.2 μm syringe filter. We also concentrated the ~10 mls of filtered media with a Millipore Amicon 100 kDa filter apparatus down to ~0.5 mls. Total RNA from media and plasma samples was purified using the miRNeasy Serum/Plasma kit (Qiagen). RT-PCR was performed using the TaqMan Small RNA Assays (Applied Biosystems) using a total RNA input concentration of 10 ng in 15 μl total reaction mixture and 1.33 μl of the RT product was used in the 20 μl total PCR mixture. The miRNeasy Serum/Plasma Spike-In Control (cel-miR-39) was used for normalization of miRNA purification. Sec-miR levels were assessed using a TaqMan MicroRNA assay (Applied Biosystems, Foster City, CA) with *in silico* designed custom primers (Assay ID: CS20SPM) specific for the mature Sec-miR sequence (AAAUGUACUGCGCGUGGAGAC). Amplification of U6 snRNA served as an endogenous control to normalize Sec-miR expression data for cells. Amplification of cel-miR-39 was used to normalize Sec-miR expression data from cell culture media and serum. All qRT-PCR assays were performed using an iCycler Real-Time PCR detection system (Bio-Rad).

### Statistics

Comparison of data between two groups was performed using an unpaired two-way student t-test. Comparison of data between more than two groups was accomplished using an ANOVA followed by a Tukey’s post-hoc multiple comparisons test. Pearson correlational analysis was performed between individual data sets. A nominal p-value <0.05 was determined to be significant for all tests.

## Results

### Secreted miRNA (Sec-miR) is detectable in the media of both transiently transfected and stably-expressing cultured cells

**Fig 1:**
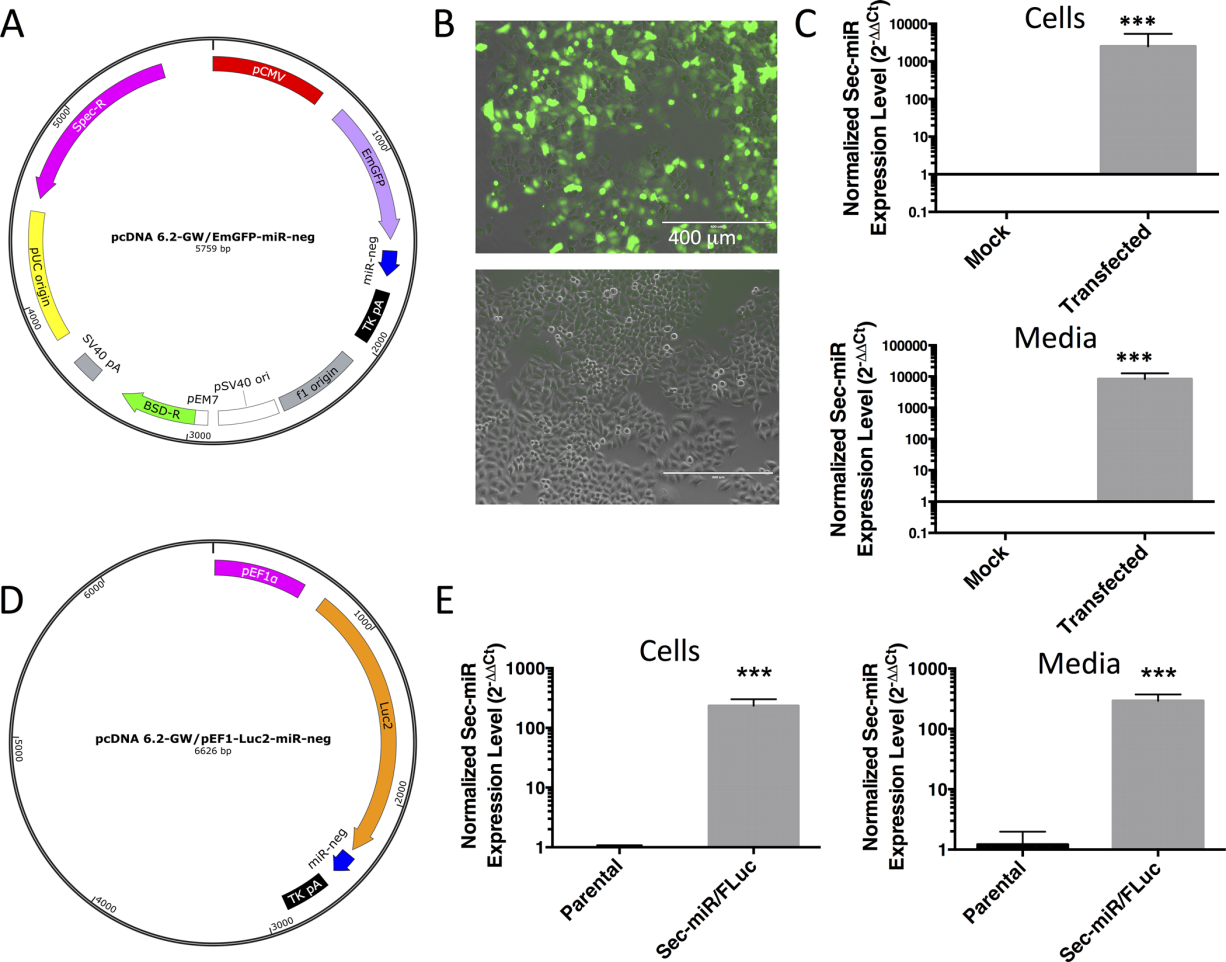
An artificial secreted miRNA reporter (Sec-miR) is detectable in the media of transiently transfected cultured cells. **A)** Vector map of pcDNA 6.2-GW/EmGFP-miR-neg. The pre-miR (miR-neg), necessary for mature Sec-miR generation, is encoded within the 3’ untranslated regions (3’-UTR) of the pCMV-EmGFP expression cassette. Spec-R–Spectinomycin resistance; BSD-R–blasticidin resistance; pUC origin–origin of replication. **B)** Fluorescence/phase images of HeLa cells 24 hours after transfection with pcDNA 6.2-GW/EmGFP-miR-neg (top) or mock-transfected (bottom). **C)** Sec-miR levels measured in HeLa cells (top) or media (bottom) transfected with pcDNA 6.2-GW/EmGFP-miR-neg (or mock transfected) 72 hours after transfection. Significantly higher Sec-miR levels were detected in transfected cells compared to mock cells in both the cell pellets (p<0.001) and the media (p<0.001). Sec-miR data from transfected cells is normalized to data from mock transfected cells and expressed as mean ± SD. **D)** Vector map of pcDNA 6.2-GW/ pEF1-Luc2-Sec-miR-miR-neg. miR-neg is encoded within the 3’ untranslated regions (3’-UTR) of the pEF1-Luc2 expression cassette. **E)** Significantly (p<0.001) higher Sec-miR levels are measured in cells and media of cells stably expressing Sec-miR/FLuc versus parental HeLa cells. Sec-miR data from Sec-miR/FLuc cells is normalized to data from parental cells and data is expressed as mean ± SD.

Our first objective was to determine whether Sec-miR could be effectively secreted/released from cells transiently expressing it. To evaluate this, we transfected HeLa cells with pcDNA 6.2-GW/EmGFP-miR-neg (Fig 1A; n = 3), the original vector co-expressing EmGFP and the pre-miRNA (132 nt), called miR-neg, that is processed to generate mature Sec-miR. We then measured Sec-miR both in cell lysates and culture media 72 hours later (Fig 1). Transfection was confirmed by the presence of EmGFP fluorescence in transfected (transfection efficiency of ~51.6%) versus mock-transfected cells (Fig 1B). Sec-miR was detectable in both the cells (p<0.001) and the media (p<0.001) of transfected cells (Fig 1C). Sec-miR signal in mock transfected cells (n = 3) was equivalent to no template control wells (i.e., assay background). Thus the Sec-miR reporter is detectable and is released/shed from HeLa cells.

We also established a HeLa cell line (Sec-miR/FLuc HeLa) that stably expressed FLuc (Luc2 gene) and our Sec-miR reporter driven by pEF1 after transfection with pcDNA 6.2-GW/pEF1-Luc2-miR-neg and clonal selection (Fig 1D). In culture, Sec-miR/FLuc cells had significantly (p<0.0001) higher levels of Sec-miR in the cells (n = 6) and media (n = 5) compared to the background levels (i.e., no expression) for parental HeLa cells in both cell lysates (n = 3) and media (n = 5) (Fig 1E).

### Sec-miR is detectable in the serum of animals bearing Sec-miR-expressing tumors and can be used as a complement to imaging reporters

**Fig 2:**
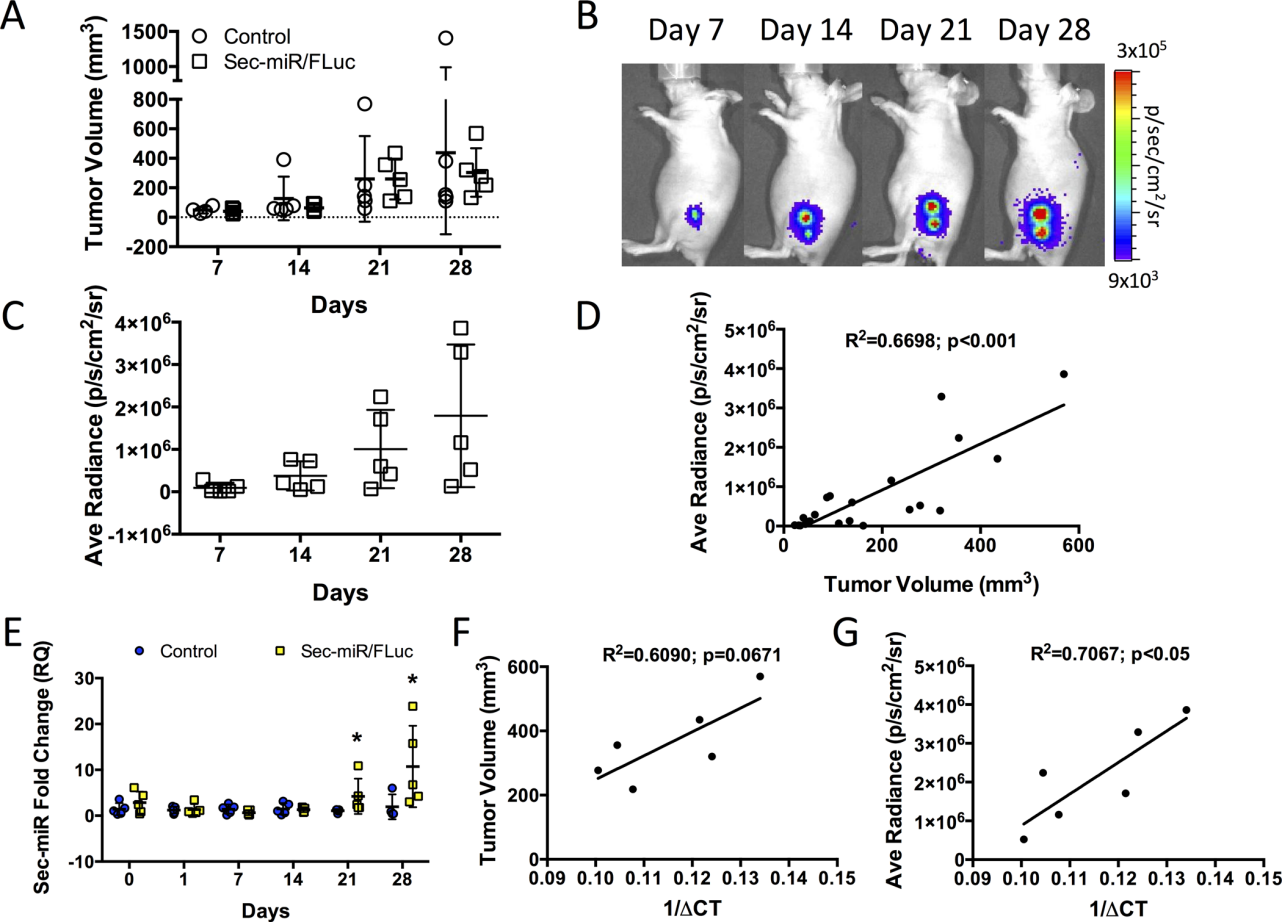
Sec-miR is detectable in the serum of animals bearing Sec-miR-expressing cells and can be used as a complement to imaging reporters. **A)** Tumor volume measurements over time of mice implanted with parental (n = 5) or Sec-miR/FLuc (n = 5) HeLa cells. Data is expressed as mean ± SD. No differences in tumor volume are seen between the two cell lines. **B)** Bioluminescence imaging (BLI) of a representative Sec-miR/FLuc HeLa mouse over time. Data is expressed as mean ± SD. **C)** Quantitative ROI tumor analysis of BLI images was performed showing the increase in average radiance over time. **D)** Correlation of tumor volume and BLI measures. **E)** Sec-miR levels in the serum over time in mice bearing parental or Sec-miR/FLuc-expressing HeLa tumors. Significantly (p<0.05) higher Sec-miR levels are detected on days 21 and 28 in Sec-miR/FLuc mice versus controls. Data from Sec-miR/FLuc HeLa mice are normalized to data from parental HeLa mice and expressed as mean ± SD. Correlation of Sec-miR levels (1/delta-Ct) and tumor volume **(F)** and FLuc BLI signal **(G)** for Sec-miR/FLuc HeLa mice.

Separately, parental and Sec-miR/FLuc HeLa cells were implanted subcutaneously into the flank of Nu/Nu mice (n = 5/group). Tumor growth was monitored over 28 days (Day 7, 14, 21, and 28) by measuring tumor volume with calipers (Fig 2A), performing BLI and doing tumor ROI analysis (Fig 2B and 2C), and measuring Sec-miR levels in blood (Fig 2E). Tumor volume increased over time for both HeLa and Sec-miR/FLuc mice at the same rate (Fig 2A). For Sec-miR/FLuc mice, BLI signal was detected at all time points (Fig 2B), increased significantly over time (Fig 2C), and tumor volume correlated with FLuc BLI signal (R^2^ = 0.669808; p<0.001; Fig 2D). Significantly (p<0.05) increased Sec-miR blood levels in Sec-miR/FLuc HeLa versus parental HeLa mice were detected by day 21 and 28 after cell implantation (Fig 2E). Data for individual Sec-miR/FLuc mice for tumor volume, FLuc signal, and Sec-miR levels are shown in S1A–S1C Fig, respectively. Qualitatively, it appeared that mice with the largest tumors tended to have the highest Sec-miR blood levels. We corroborated this by demonstrating a trend towards a positive correlation between Sec-miR levels in Sec-miR/FLuc mice after day 21 and tumor volume (R^2^ = 0.6090, p = 0.0671; Fig 2F), and a significant positive correlation to FLuc signal (R^2^ = 0.7067; Fig 2G), indicating Sec-miR levels can be used to complement reporter imaging measures of cell number. Correlational analysis was only performed using Sec-miR data from Sec-miR/FLuc mice that was greater than 2 standard deviations above the mean Sec-miR levels in control mice (6 of 10 values from days 21 and 28).

### Expression of multiple Sec-miR copies within a construct can amplify reporter secretion from cells

**Fig 3:**
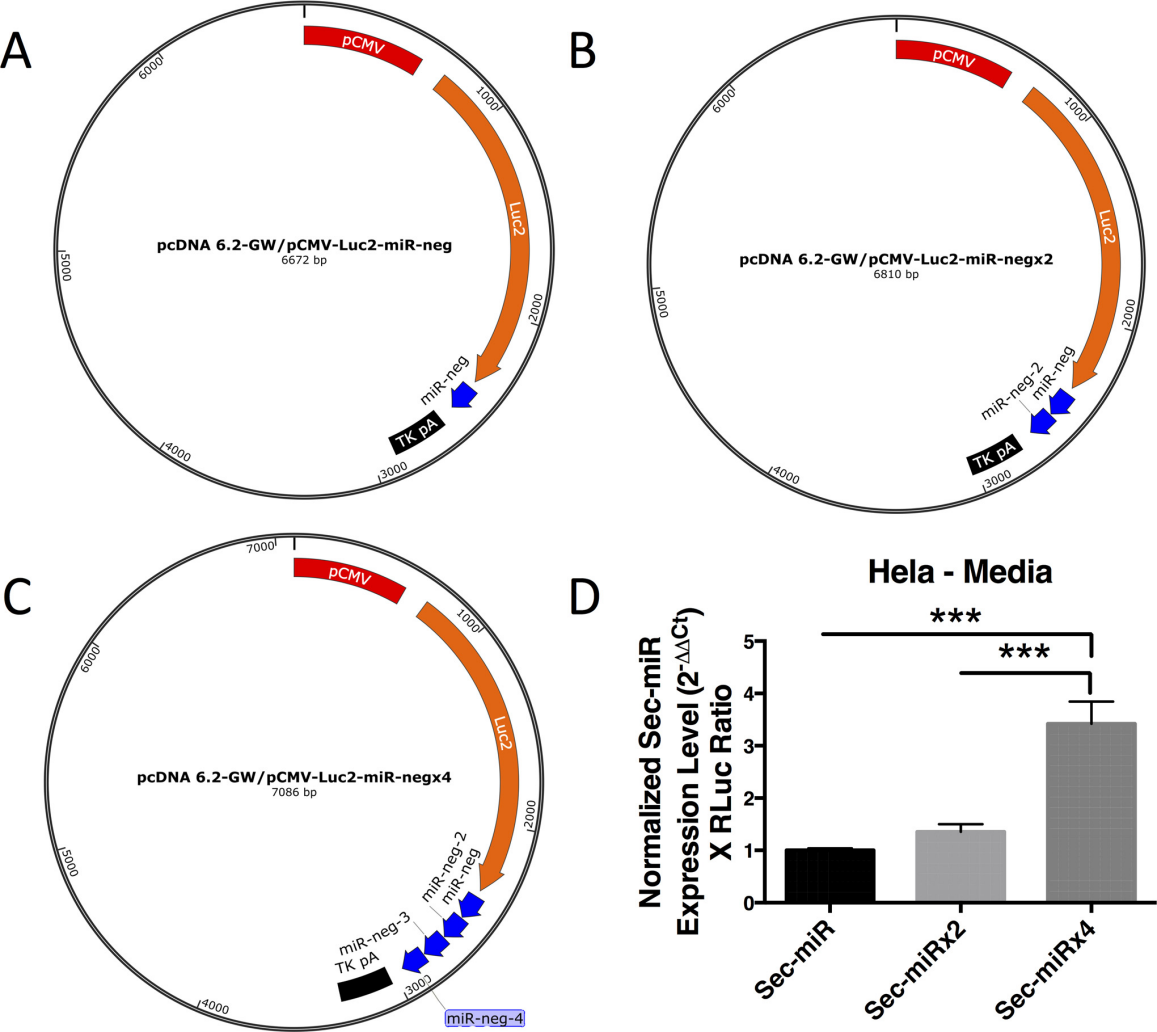
Expression of multiple Sec-miR copies within a construct can amplify reporter secretion from cells. **A)** Vector maps of Sec-miR-expressing constructs containing 1, 2, or 4 copies of miR-neg (A to C). **B)** Sec-miR levels in cell culture media in HeLa cells (left to right) after transient transfection with the vectors shown in **A-C** and a Renilla luciferase (RLuc) expressing plasmid to control for transfection efficiency. All raw qPCR data was normalized to Renilla luciferase (RLuc) activity and then across groups to values in cells transfected with the 1 miR-neg copy vector. Data is expressed as mean ± SD. All constructs resulted in detectable levels of Sec-miR in the media and significantly higher levels were detectable for the construct containing 4 copies of miR-neg versus the other constructs (p<0.01).

While the levels of Sec-miR in mouse blood were relatively low, we hypothesized it would be possible to amplify secretion from cells by tandemly expressing (or chaining) more than one Sec-miR in the same construct (Fig 3). This should not drastically alter the size of the construct due to the small size of the miR-neg pre-miRNA (132 nt). We show that increasing the number of copies of miR-neg within the construct from 1 to 4 (Fig 3A–3C) significantly (p<0.01) increased the Sec-miR levels found in the media of transiently transfected HeLa cells (Fig 3D). Interestingly, the 3 extra copies of Sec-miR led to an ~4-fold change in Sec-miR levels in the media and suggests that our amplification strategy may be linear.

Overall, we demonstrate Sec-miRs can be secreted by cancer cells and exist in a stable form that allows their detection in fluids such as cell culture media and serum, highlighting their potential as a new class of secreted reporter.

## Discussion

Imaging reporter genes have been extremely useful for tracking various transplanted cell populations such as cancer cells, stem cells, and immune cells in pre-clinical models, and have recently been successfully translated into the clinic [11]. Alternative or additional use of secreted reporters could enable higher throughput and more frequent monitoring of overall cell survival using relatively cheap blood-based (or other biofluid-based) assays. Here we have successfully developed an RNA-based secreted reporter and demonstrate that it can be used in concert with imaging reporter genes to monitor the proliferation of transplanted cells in living subjects. This reporter adds to the minimal collection of secreted reporters available and could find use for many diverse applications.

Secreted RNA reporters have not been previously described. This most likely stems from the fact that bare RNA is relatively unstable in the blood due to the high levels of RNAse present. As miRNAs exist in a stable form that is protected from degradation, and have shown promise as serum disease biomarkers, we reasoned it would be possible to explore them as a new class of reporter genes with a secretable product. Interestingly, other RNA species (e.g., long non-coding RNAs, etc.) also exist in exosomes or other extracellular vesicles and may also be useful as reporter genes. Many different miRNAs have been found in the blood, but we chose not to utilize an endogenously expressed miRNA, as this would lead to a background signal. Nor did we utilize a miRNA that targets any gene, as we wanted our reporter to remain “neutral” and not promote any endogenous mRNA translational regulation. Future work will include a deeper look at other indications of biological effects of our reporter, such as repression of endogenous miRNA regulation capabilities, as well as a study of its localization in blood to within exosomes or bound to protein complexes such as Argonaute2 [36].

One of the current limitations of our reporter was the relatively high tumor volume (i.e., high number of Sec-miR-expressing cells) needed to enable detection of Sec-miR in the blood (Fig 2). At present, established protein-based reporters (e.g., SEAP and GLuc) are expected to be more sensitive [13, 20]. We hope to address these sensitivity issues by exploring several avenues including: generating clonal populations expressing higher levels of Sec-miR prior to transplantation into animals; tandem chaining many miR-neg copies together (up to 32 is approximately the size of the SEAP reporter gene) as we have provisionally explored with a tandem repeat of 4 units (Fig 3); the use of more sensitive detection platforms than analog real-time PCR such as droplet digital PCR (ddPCR) [40] (40)[40]; and/or incorporation of improved ways to shuttle Sec-miRs out of cells. It is also possible to explore alternatively designed Sec-miR sequences that will be more readily secreted out of cells [37]. Certain cell types may also secrete the Sec-miRs more readily than others, so alternative cell types, particularly therapeutic cell populations (e.g., stem cells or immune-cell based therapies), will be engineered to express optimized reporters to test feasibility and *in vivo* sensitivity. Continued unraveling of the mechanisms that regulate miRNA shedding/release from cells into the extracellular milieu and into the blood could be leveraged to transform Sec-miRs into sensitive blood-based reporters.

An important consideration for the use of Sec-miRs, as is the case for most secreted reporter genes, is the half-life of miRNAs in circulation of more than 24 hours [26, 41]. This could cause errors in the correlation of labeled living cells to secreted biomarker levels due to the persistent presence of the marker shed from cells that could have already undergone apoptosis or necrosis. We do not think this would be a substantial issue due to our proposal of the combined use of Sec-miR together with an imaging reporter to follow cellular trafficking. As we improve our secretion rates as well as sensitivity of the reporter model this issue will be carefully considered in correlating the circulating concentration to live cell number. An interesting finding is that our Sec-miR levels significantly correlated with BLI measures but not tumour burden. BLI is a direct measure of the number of viable Sec-miR-expressing cells, whereas tumor volume can measure alive and dead tissue, so it is not unreasonable for us to obtain this result. Modeling of biomarkers shed into circulation takes into consideration this issue and our lab is working on modeling the input of secreted biomarkers in blood from dead cells [42].

Another issue we encountered is the non-specific binding of our Sec-miR primers in the samples from the media or plasma of non-transfected control cells, which gave us non-zero Ct values. We attempted different primer sets synthesized *in silico* and used the primer set that gave us the least amount of background compared to no-template controls. This non-specific binding was not due to the primer dimerization but due to some unexplained binding with the sample, as determined during further studies using a digital droplet PCR instrument (data not shown). Importantly, both the no-template controls and control cells gave relatively the same Ct values. Further procedure optimization, design and testing of other Sec-miR sequences, or design of primer/probe sets could decrease this binding, and increase the difference of Sec-miR values between the control and transfected/stable cells. This issue is occasionally experienced in miRNA qRT-PCR, is a particular issue for our study due to the low concentration of the Sec-miR in the plasma samples, and will be addressed in the future with the above efforts. Improved secretion rates of Sec-miR *in vivo* could also minimize this issue.

One potential advantage of Sec-miR reporters over protein reporters is their modular nature. It should be possible to modify both the coding sequence and paired qRT-PCR assay primers to generate multiple unique Sec-miR reporter systems. These systems could then be multiplexed together to tag different cell populations with a particular Sec-miR sequence and track the survival of more than one cell population at the same time. Alternatively, the different miR sequences could be linked to different non-constitutive promoters in order to monitor intracellular events that will then lead to a unique Sec-miR.

Secreted reporters also have utility outside of cell tracking. For instance, we have recently developed non-viral gene vectors called tumor activatable minicircles expressing a secreted reporter (i.e. SEAP) driven by the tumor-specific Survivin promoter [43]. Upon system administration, this system was able to differentiate tumor-bearing from normal mice using a simple blood test. Once Sec-miRs are more fully developed and optimized we could include them into tumor-activatable minicircle systems.

To our knowledge, this is the first work exploring the development of an RNA-based blood reporter and its use as a complementary tool to imaging reporters for cell tracking. We demonstrate the capability of this system for monitoring of the number of viable cells in a preclinical setting. The sensitivity of the RNA reporter system was found not to be as sensitive as BLI in living mice, however we hypothesize our blood reporter will be more sensitive than clinically-relevant PET reporter imaging. Future work will test this hypothesis, in addition to exploring various optimization strategies and potential applications of Sec-miR reporters.

## Supporting Information

S1 Fig Tumor volume, average BLI signal, and Sec-miR serum levels of individual mice over time. Individual Sec-miR/FLuc HeLa mouse measures over time for tumor volume (**A**), average radiance from BLI images (**B**), and serum Sec-miR fold change compared to control (**C**). Note the mice with the largest tumors in **A** and **B** tended to have the highest Sec-miR values in the serum in **C**.
(PDF)
