# Supplementary material for: Artificial MicroRNAs as Novel Secreted Reporters for Cell Monitoring in Living Subjects
Source: PLoS One. 2016 Jul 21;11(7):e0159369. doi: 10.1371/journal.pone.0159369 (PMC4956193; doi:10.1371/journal.pone.0159369)

Supporting Information

S1 Fig. Tumor volume, average BLI signal, and Sec-miR serum levels of individual mice over time.

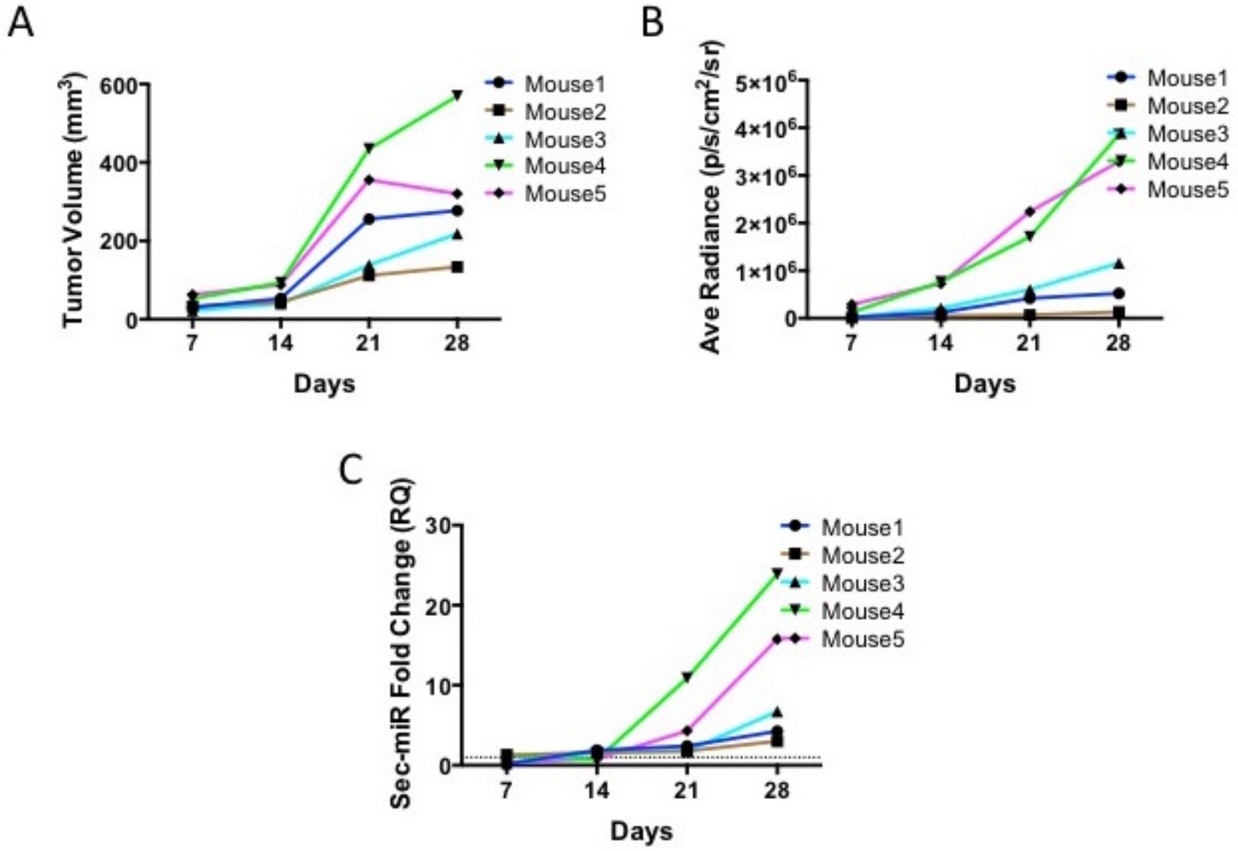

Supplement: S1 Fig — Individual Sec-miR/FLuc HeLa mouse measures over time for tumor volume (A), average radiance from BLI images (B), and serum Sec-miR fold change compared to control (C). Note the mice with the largest tumors in A and B tended to have the highest Sec-miR values in the serum in C. (PDF) [file pone.0159369.s001.pdf]
